# Alteration in the Function and Expression of SLC and ABC Transporters in the Neurovascular Unit in Alzheimer’s Disease and the Clinical Significance

**DOI:** 10.14336/AD.2019.0519

**Published:** 2020-03-09

**Authors:** Yongming Jia, Na Wang, Yingbo Zhang, Di Xue, Haoming Lou, Xuewei Liu

**Affiliations:** ^1^Department of Neuropharmacology, College of Pharmacy, Qiqihar Medical University, Qiqihar, China; ^2^Department of Pathophysiology, Basic Medical Science College, Qiqihar Medical University, Qiqihar, China; ^3^College of Pathology, Qiqihar Medical University, Qiqihar, China; ^4^Department of Medicinal Chemistry and Chemistry of Chinese Materia Medica, School of Pharmacy, Changchun University of Chinese Medicine, Changchun, China

**Keywords:** blood-brain barrier, ATP-binding cassette transporters, solute carrier, Alzheimer’s disease

## Abstract

The neurovascular unit (NVU) plays an important role in maintaining the function of the central nervous system (CNS). Emerging evidence has indicated that the NVU changes function and molecules at the early stage of Alzheimer’s disease (AD), which initiates multiple pathways of neurodegeneration. Cell types in the NVU have become attractive targets in the interventional treatment of AD. The NVU transportation system contains a variety of proteins involved in compound transport and neurotransmission. Brain transporters can be classified as members of the solute carrier (SLC) and ATP-binding cassette (ABC) families in the NVU. Moreover, the transporters can regulate both endogenous toxins, including amyloid-beta (Aβ) and xenobiotic homeostasis, in the brains of AD patients. Genome-wide association studies (GWAS) have identified some transporter gene variants as susceptibility loci for late-onset AD. Therefore, the present study summarizes changes in blood-brain barrier (BBB) permeability in AD, identifies the location of SLC and ABC transporters in the brain and focuses on major SLC and ABC transporters that contribute to AD pathology.

## 1. Introduction

**Figure 1.**
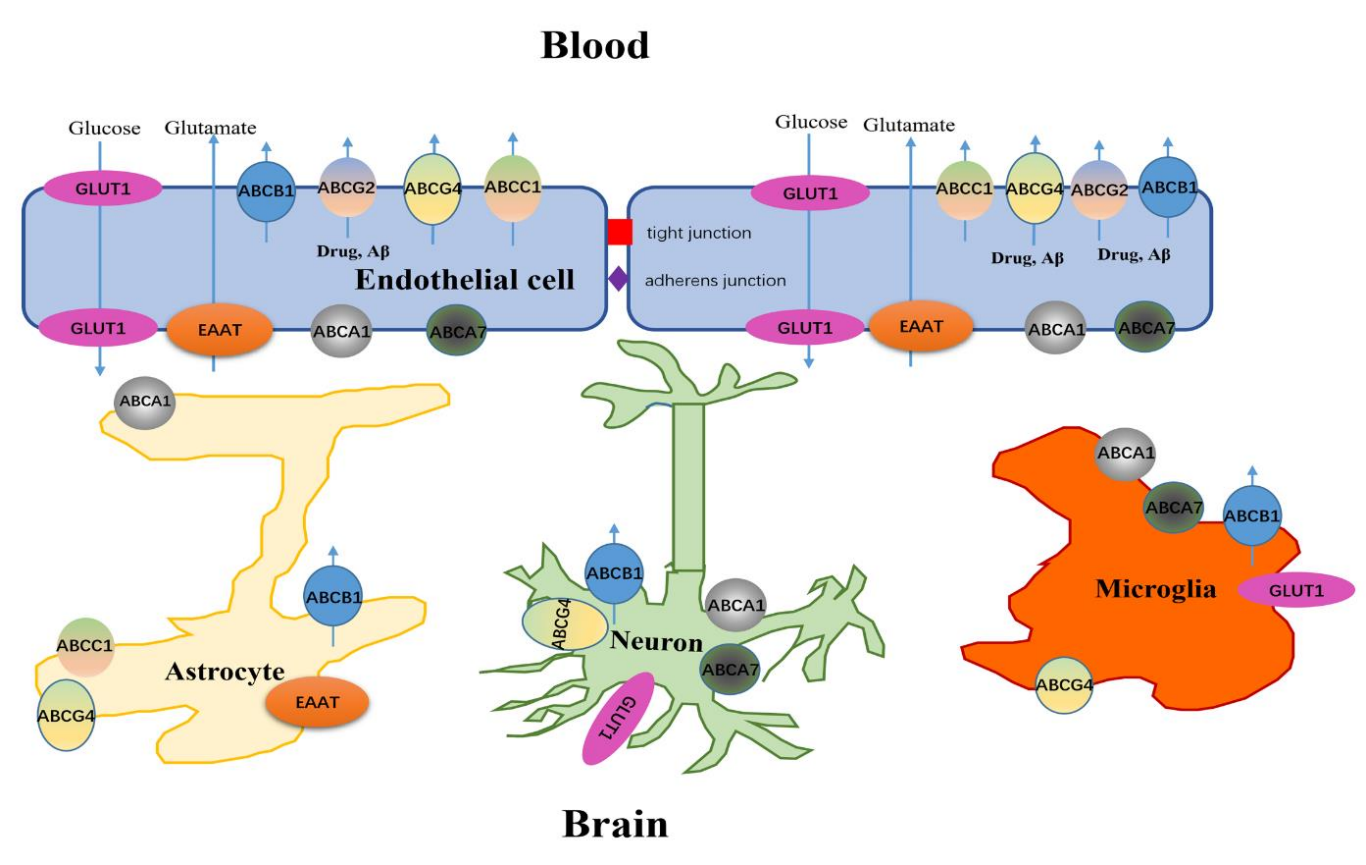
SLC and ABC transporters in the NVU.

Central nervous system (CNS) barriers are vital to the brain microenvironment for the regulation of neuronal functions, including nutrient transport and protection of the brain from toxins [1]. Brain barriers include the blood-brain barrier (BBB) and blood-cerebrospinal fluid (CSF) barrier (BCSFB) [2]. BBB, a semipermeable border, is composed of brain microvascular endothelial cells that are sheathed by pericytes, perivascular astrocytic end-feet, neurons and microglia, which constitute a functional unit, namely, the neurovascular unit (NVU). The BCSFB, a fluid-brain barrier, is composed of choroid plexus epithelial cells whose primary function is to secrete CSF; these cells are interconnected by unique apical tight junctions (TJs) [3]. Previous studies found that transporters expressed in endothelial cells could be involved in the influx of essential nutrients such as amino acids and glucose and in the efflux of endogenous toxins such as amyloid-beta (Aβ) and exogenous substance [4]. Brain transporters in the BBB can be classified as uptake transporters (including the solute carrier (SLC) superfamily) and efflux transporters (including the ATP-binding cassette (ABC) superfamily). These transporters are considered to play key roles in the treatment and pathogenesis of CNS disorders such as AD (Fig. 1) [5, 6]. SLC transporters in the brain, including *SLCO*, *SLC22A*, *SLC28A* and *SLC29A*, can transport substrates from blood to the brain to meet the high energy and nutrient demands of the brain; such substrates include organic cations and anions, peptides, steroids and drug conjugates [7]. However, as a cellular defense system, ABC transporters extrude metabolic wastes from the cytoplasm to the blood and prevent most xenobiotics from entering the brain. ABC transporters, classified into seven subfamilies from ABCA to ABCG, show extensive overlaps in substrate specificity [8]. At the same time, ABC transporters are expressed widely in endothelial cells, neurons and glial cells of the brain. Emerging evidence suggests that abnormal NVU transporter expression is correlated with CNS diseases, including glioblastoma, Parkinson’s disease and AD [9, 10]. Furthermore, loss of BBB integrity and transporter dysfunction affect the entry or efflux of compounds, disrupting CNS homeostasis and thereby exacerbating CNS diseases, including AD and stroke.

AD, a typical neurodegenerative disorder, is characterized by the presence of amyloid plaques and neurofibrillary tangles. It is also associated with microvascular dysfunction and/or degeneration in the brain [11]. It is well known that microvessels are key sites for the exchange of substances, such as nutrients, energy substrates and the brain’s supply of oxygen, between the brain and circulating blood [12]. Microvascular pathology causes the disruption of BBB integrity. Furthermore, studies have reported that the BBB is significantly affected in different models of AD-related microvascular pathology [13]. Moreover, destruction of BBB integrity diminishes the clearance of neurotoxic molecules such as soluble Aβ from the CNS and consequently leads to accumulated Aβ in the brain [14]. Some transporters, especially ABC transporters, can mediate Aβ homeostasis in the brain and are key players in AD pathology [15]. This review summarizes changes in BBB permeability in AD, identifies the location of SLC and ABC transporters in the brain and focuses on major SLC and ABC transporters that contribute to AD pathology.

## 2. BBB breakdown in AD

BBB permeability can be influenced by physicochemical factors, including the molecular weight, lipid solubility and surface area of molecules. In general, most small-molecule drugs cannot pass through the BBB. Only certain small molecules that are lipid soluble with less than a 400 Da molecular weight can pass through BBB. In addition, physiological factors that influence BBB permeability include efflux transporters such as ABCB1, plasma protein binding, cerebral blood flow and an enzymatic surveillance system [16, 17].

BBB permeability was found to change in patients with mild cognitive impairment (MCI) and AD by using an advanced dynamic contrast enhanced (DCE)-MRI method [18]. Nevertheless, the cause and progression of BBB breakdown and dysfunction in AD are still not fully known. Numerous transgenic AD models have been used to explore the mechanism of BBB breakdown. For instance, studies have shown that APP^Sw/0^ mice, which overexpress cellular APP and exhibit Aβ overproduction, finally develop BBB breakdown. BBB breakdown can be demonstrated by capillary leakages of blood-derived molecules, including albumin and IgG, loss of endothelial tight junction proteins and endothelial cell and pericyte degeneration in AD [19]. Similarly, in presenilin1 (PSEN1) and Tau transgenic models, BBB leakage of Evans blue, perivascular deposits and major cerebrovascular pathology indicates BBB breakdown. The strongest genetic risk factor for AD is the E4 variant of apolipoprotein E (ApoE), which can affect Aβ clearance and tau-related neurodegeneration [20]. Furthermore, in Apoe^-/-^ mice, BBB breakdown is presented by loss of TJs, loss of perivascular pericytes, and hemosiderin deposits [21]. Aberrant expression of brain transporters can also be seen in transgenic models [22, 23]. In *in vitro* BBB models, Aβ oligomers induce the disruption of tight junction scaffold proteins via the receptor of advanced glycation end-products (RAGE)-mediated autophagy pathway in capillary endothelial cells of the murine brain (bEnd.3), leading to BBB breakdown [24].

### 2.1. BBB junctional molecules in AD

TJs and adherent junctions (AJs) between BBB endothelial cells are a highly specialized structure with barrier integrity that can limit paracellular permeability and thus maintain brain homeostasis [25]. Molecular components of TJs contain membrane proteins, such as claudins and occludin, and cytoplasmic proteins, such as zonula occludens-1 (ZO-1), which are involved in the construction of TJs at the BBB [26]. Similarly, the AJ also plays a key role in endothelial permeability. The major proteins of AJs are VE-cadherin, catenin and platelet endothelial cell adhesion molecule (PECAM) [27]. The AJ proteins directly regulate the paracellular route and alter transcellular exchange and affect certain enzymes and transporters in the BBB [25]. Recent studies have shown that BBB breakdown in the hippocampus can be found in AD patients at the early stage [18]. Consistent with these findings, in 20 independent postmortem tissues from AD patients, BBB breakdown was observed, accompanied by brain capillary leakages and loss of BBB tight junctions [28]. Changes in junctional protein expression influence a variety of protein functions. Under normal conditions, claudin-5 and ZO-2 display a continuous distribution along the plasma membrane in cell-cell contacts, but brain endothelial cells treated with Aβ_1-42_ for 3 days cause TJ protein relocation to the cytoplasm, decreased occludin expression and altered BBB integrity, contributing to the pathogenesis of AD [29]. However, changes of TJs have impaired BBB against small molecules (<800 Da) but not larger molecules. Chemicals such as TJ modulators are used for drug delivery to neuronal regions [22, 30-31]. This suggests to us a new way to deliver potential drugs across the BBB into the CNS to treat neurodegenerative diseases.

### 2.2. Pericyte degeneration in AD

Pericytes are a major component of the NVU. As BBB gatekeepers, they transport nutrients and waste molecules between the blood and the brain interstitial fluid [13, 32]and help to regulate blood flow. Mounting evidence has shown that pericytes are related to BBB pathology in AD. Pericyte degeneration and loss have been found in AD patients by postmortem analysis [33, 34]. Moreover, Aβ deposition around brain capillaries can promote ROS overproduction in pericytes and induce toxicity in pericytes, leading to BBB breakdown [35, 36]. Progressive BBB breakdown is displayed in pericyte-deficient mice, leading to hippocampal neuron loss and behavioral deficits [37]. Interestingly, pericytes are involved in Aβ clearance in the brain [38]. Pericytes clear Aβ aggregates via an LRP1-dependent apoE isoform-specific mechanism in *APP*^Swe/0^ mice [39]. In addition, mesenchymal stem cell-derived pericyte implantations have the capacity to reduce Aβ deposition in the hippocampus of APP transgenic mice and related pathology [32]. These studies suggest that pericytes may be a potential therapeutic target for controlling Aβ clearance in AD.

## 3. SLC transporters and AD

### 3.1. Glutamate transporters

Glutamate transporters, members of the SLC1 and SLC17 family, comprise two subclasses, namely, the excitatory amino acid transporter (EAAT) family and vesicular glutamate transporter (VGLUT) family, regulating glutamate homeostasis at the synapse and affecting excitotoxic neuronal damage in certain neurodegenerative diseases such as AD [40]. EAAT1 (SLC1A3) and EAAT2 (SLC1A2) are expressed in astrocytes and are responsible for 5% and 90% of the glutamate transport in the CNS, respectively [41, 42]. Studies have shown that pathology-specific EAAT2 splice variants in the brains of AD patients obtained by autopsy and glutamatergic dysfunction are involved in the pathophysiology of AD and occur in the early stage of AD [43]. The accumulation of excessive extracellular glutamate has been considered a risk factor in neurodegenerative diseases [44]. In addition, memantine, as a typical metabotropic glutamate receptor antagonist, is used in the symptomatic treatment of AD. Therefore, EAATs might be an intriguing therapeutic target [44]. However, there are conflicting reports of EAAT2 expression in different animals with AD. In transgenic mice overexpressing mutant human APP, the expression and activity of EAAT1 and EAAT2 protein were found to be decreased in the mouse brain [45]. Similarly, in APP transgenic mice, the researcher found that EAAT2 protein expression decreased in the cortex and hippocampus, leading to a drastic glutamate reuptake activity decrease [46]. However, in double (APP/PS1) and/or triple transgenic (3xTg-AD) mice, EAAT2 protein expression did not change over the lifetime of the mice [47]. These conflicting results may be related to the EAAT2 location and methods of qualitative analysis, and further research is needed to verify the findings. Because of glutamate homeostasis in AD, researchers have focused on developing or screening new compounds that can activate EAAT protein expression, providing a beneficial effect in AD patients.

### 3.2 Glucose transporter

Glucose transporters (GLUTs) can be divided into two major families, namely, the SLC2A family (GLUTs) and SLC5A family (SGLTs) [48]. The two families have 14 isoforms (GLUTs1-14) and 12 isoforms (SGLTs 1-12). Endothelial cells at the BBB can transport glucose rapidly to support the glucose requirements of the brain [49]. GLUTs are cell type-specific and are affected by disease conditions. GLUT1 is predominantly expressed at both the luminal and abluminal membranes in the endothelium of the BBB [50]. Glucose in the extracellular space is permeated into astrocytes, microglia and neurons through different GLUTs [51]. GLUT1 and GLUT3 expressed in neurons are major glucose transporters in the regulation of glucose homeostasis in the brain [52]. The majority of brain glucose is used in the formation of ATP energy to maintain cognitive functions [53]. Glucose metabolism abnormalities play a crucial role in the pathogenesis of AD [54].

In sporadic AD, ATP produced by glucose metabolism in the brain declines by 50% [55]. Recent research has found both altered glucose metabolism in the brain and reduced glucose transporter expression associated with AD [56, 57]. Evidence has shown that glucose transport is reduced in AD patients’ brain regions, such as the cortex, hippocampus, and cerebral microvessels [58]. Furthermore, GLUT1 and GLUT3 protein concentrations in the cerebral cortex are reduced significantly in AD patients [59]. Moreover, GLUT1 deficiency leads to early BBB breakdown in *Slc2a1^+/-^* mice, and decreased GLUT1 protein expression is found in the brain microvessels of patients with AD and cognitive impairment [60, 61]. In *Slc2a1^+/-^* transgenic mice, rapid BBB breakdown can be seen following secondary neurodegeneration caused by acceleration of Aβ, suggesting that GLUT1 plays a critical role in AD [60]. Moreover, GLUT1 deficiency in the endothelium of mice with overexpressed APP exacerbates cerebral microvascular degeneration and BBB breakdown, leading to accumulated A? and behavioral deficits [60]. Indeed, reduced GLUT1 at the BBB has been reported to be one of the earliest features of AD pathophysiological events and symptoms [62]. As a consequence, a number of studies suggest that impaired glucose metabolism and decreased glucose transporter expression are causative factors in the progression of AD. However, the therapeutic potential of targeting glucose transporters has not been defined and requires further research. GLUT1 could be a molecular marker for the onset of AD and a therapeutic target for preventing, delaying or treating AD.

### 3.3 Nucleoside transporters

Nucleoside transporters can be classified into two families: the SLC28A family (concentrative nucleoside transporters, CNTs) and the SLC29A family (equilibrative nucleoside transporters, ENTs) [63]. The two SLC families have 3 isoforms (CNTs1-3) and 4 isoforms (ENTs 1-4). CNTs are primarily expressed in epithelial tissues, such as the kidney and liver. CNT2 and CNT3 genes are highly expressed in human brain regions, such as the hippocampus and medulla oblongata. Moreover, CNT2 has been detected in rat brain endothelial cells [64]. However, CNTs1-3 expression levels are below the limit of quantification in proteomic analyses of humans, indicating that these proteins may not be involved in the transport of nucleosides in humans [65].

ENT proteins show a wide tissue distribution in the liver, kidney, intestine and brain. They are highly expressed in the plasma membrane and the frontal and parietal cortices [66]. ENT1 is the main regulator of homeostatic maintenance of adenosine levels depending on the concentration gradient of the membrane. Recent studies have found that J4, a small adenosine analogue, can inhibit ENT1 function and further prevent the decline in spatial memory in APP/PS1 mice. Furthermore, the author demonstrated that chronic treatment with J4 improves impaired basal synaptic transmission and abnormal neuronal plasticity-related signaling pathways [67]. ENTs could mediate the transport of several nucleoside drugs into the brain. These results suggest that ENT1 is a therapeutic option in AD treatment.

### 3.4 Other uptake transporters and AD

L-alpha amino acid transporters (LATs), members of SLC7 and SLC43, comprise four isoforms: SLC7A5 (LAT1), SLC7A8 (LAT2), SLC43A1 (LAT3), and SLC43A2 (LAT4), regulating large and small amino acid homeostasis in the brain [68]. LAT1 and LAT2 are expressed on luminal and abluminal surfaces of the BBB, and LAT1 can transport several CNS-active drugs, such as gabapentin, pregabalin, L-DOPA, and methyldopa [68-70]. However, LAT1 mRNA and protein levels in astrocytes of AD transgenic primary mice are not changed compared with those of wild-type mice. In addition, LAT1 function at the BBB in APP/PS1 transgenic mice is also unchanged compared with that in nontreated wild-type mice [71].

Organic anion transporting polypeptides (OATPs) are SLCO transporters with 11 isoforms identified in humans. OATP transporters are widely expressed in many tissues, such as the liver, kidney, placenta, and brain [72]. OATP1A2 (Oatp1a4/Slco1a4 in mice) and OATP1C1 have been found at the BBB [73]. Moreover, OATP1A2 plays a key role in drug delivery to the brain and can mediate the uptake of drugs such as digoxin and DPDPE in the BBB [74]. Recent studies have found that rosuvastatin and taurocholate, which are inhibitors of Oatp1a4, decrease the Aβ concentration in mouse brains using *in situ* brain perfusion and have further investigated the interaction between Oatp1a4 and Aβ by coimmunoprecipitation, indicating that Oatp1a4 at least partly mediates Aβ uptake [75]. Whether OATP1A2 and/or Oatp1a4 expression levels are changed or whether pathological stressors can change OATP expression in the BBB requires further research.

Organic anion transporters (OATs) and organic cation transporters (OCTs) belong to the SLC22 family and are expressed in many tissues, including the small intestine, kidney, liver, placenta, and brain capillaries. Under normal conditions, OAT expression shows distinct differences at the BBB, and OAT1 and OAT3 expression are below the limit of quantification in human brain microvessel endothelial cells and astrocytes [65, 76]. However, in an OAT1-deficient AD mouse model, learning- and memory-related behavior deficiency, reduced LTP levels and higher soluble Aβ concentrations in the early stage were increased compared with the corresponding levels in control tg2576 mice, suggesting that OAT1 may affect the AD process [77]. In addition, Oat3 and Oct2 protein expression levels were increased 1.3- and 1.4-fold, respectively, in the kidneys of APP/PS1 transgenic mice compared with those in wild-type mice, which may have affected drug elimination in this AD model [78].

OCT1 and OCT2 are mainly expressed on the luminal surface of brain capillary endothelial cells isolated from the brain cortex of humans, as analyzed by using confocal microscopy, suggesting that these two isoforms mediate the uptake of substrates across the BBB into the brain [79]. Unfortunately, to date, no further research has focused on whether OCT expression at the BBB changes.

## 4. ABC transporters and AD

### 4.1. ABCB1

ABCB1, also known as P-gp, is widely expressed in barrier and excretory tissues, providing effective protection against harmful nonpolar therapeutic drugs and xenobiotics. It was first detected at vascular endothelial surfaces of the human BBB in 1989 [80]. It is clear that ABCB1 is also expressed in pericytes, astrocytes, the choroid plexus and neurons [81]. ABCB1 of the BBB plays a central role in the occurrence and development of AD [82]. Various studies have shown that ABCB1 expressed by the BBB can regulate the transport of endoxenobiotics through the encephalon [83]. In 2001, it was first reported that Aβ interacts with ABCB1, by using ABCB1-overexpressing HEK293 cells, which provided strong evidence that ABCB1 is an Aβ transporter [84]. Furthermore, a clinical discovery provided evidence that ABCB1 function, which can be assessed using (R)-[^11^C] verapamil and positron emission tomography (PET) *in vivo*, was decreased in AD patients compared with age-matched healthy controls [85]. Similarly, in an *in vivo* study, ABCB1 deficiency at the BBB led to Aβ deposition in a P-gp deficient null mouse model. Furthermore, compared with P-gp wild-type mice, the levels of brain Aβ and enhanced Aβ deposition were increased in P-gp-deficient null mice, establishing a direct link between P-gp and Aβ metabolism *in vivo* [86]. Moreover, compared with APP/PS^+/-^ P-gp ko mice, a significant reduction of Aβ levels was found in APP/PS^+/-^ P-gp *wt* mice, suggesting that upregulating P-gp might be a valid approach to reduce Aβ levels in the brain [87]. In 243 nondemented human brain tissues, Aβ deposition in the vessel walls was found in blood vessels with low ABCB1 protein expression using an immunohistochemical method in the medial temporal lobe, while those with high ABCB1 protein expression showed low deposition of Aβ, suggesting that P-gp may influence the elimination of Aβ from the brain [88]. Conversely, when porcine brain microvascular endothelial cells (PBECs) were treated with Aβ_42_ for 48 h, βABCB1 protein expression and ABCB1 activity were reduced significantly [89]. Additionally, studies have demonstrated that ABCB1 protein levels at the BBB decline during normal aging, which is positively correlated with the accumulation of Aβ in AD [90]. Pro-inflammatory cytokines, including TNF-α, IL-1β, and IFN-γ, have been found in models of AD induced by Aβ, and these cytokines can downregulate ABCB1 mRNA and protein levels, interrupting the negative feedback loop between Aβ and ABCB1 [91]. It demonstrated that oxidative stress (OS) can increase ABCB1 expression and activity at the BBB endothelium in primary cultured rat brain endothelial cells [92]. However, conflicting data exist regarding the contribution of ABCB1 to the clearance of Aβ. For instance, in MDCK cells stably transfected with ABCB1 transported little transcytosed radiolabeled [^125^I]Aβ from the basolateral to the apical compartment, and the Aβ clearance quotient showed no significant difference in MDCK-P-gp and MDCK-Parental monolayers, suggesting that increased ABCB1 expression is sufficient to promote Aβ transcytosis [93]. Similarly, when rats were pretreated with ABCB1 inhibitors (quinidine and/or verapamil), the amount of Aβ crossing the BBB in rats was not changed [94]. Collectively, the paradoxical results can be explained by using different cell lines in the above studies and animal models, showing that some physiological changes may affect the accumulation of Aβ.

Researchers have found a new therapeutic treatment or drug targeting ABCB1 to treat AD. *In vivo* studies have shown that ibuprofen treatment can restore decreased ABCB1 mRNA and protein expression in APP/PS1 mice [95]. The study found that Huperzine A (HupA) is a substrate of P-gp. HupA extracted from Huperzia serrata is a potent inhibitor of acetylcholinesterase (AChE). HupA has been used in the treatment of AD by targeting central nicotinic and muscarinic receptors and exerts neuroprotective properties by producing a potent anti-inflammatory response [96]. In *Abcb1*-deficient mice, the brain-to-plasma concentration ratio of Huperzine A was significantly increased. The results further suggested that P-gp can mediate Huperzine A distribution in the brain distribution [97]. Similarly, 1,1’-([1,1’-biphenyl]-4,4’-diyl)bis(3-(piperidin-1-yl)propan-1-one)dihydrochloride (DL0410), a novel synthetic dual AChE/butyrylcholinesterase (BuChE) inhibitor for AD treatment, showed multitarget properties for AD treatment, such as improving cognitive deficits, enhancing synapse loss, inhibiting the activity of cholinesterase and reversing the plaque load caused by Aβ [98-100]. P-gp mediated DL0410 transport in Caco-2 and MDCK-MDR1 cells, suggesting that further efficacy and safety should be considered in drug-drug interactions in AD treatment [101].

### 4.2. ABCG2

ABCG2, known as breast cancer resistance protein (BCRP), is highly expressed in BBB endothelial cells and plays protective roles in blocking the absorption of xenobiotics [102]. Mounting evidence has shown that ABCG2 mediates Aβ transport in brain endothelial cells. *In vitro* studies showed that ABCG2 can mediate the cellular efflux of Aβ_40_ in HEK293 cells stably transfected with human ABCG2. In addition, the study further found that Aβ uptake in *Abcb1*-deficient mice can be blocked by GF120918 (a dual inhibitor of Abcb1 and Abcg2) by using an *in situ* brain perfusion technique, suggesting that Abcg2 expressed in BBB is involved in the transport of Aβ_40_ [103]. Furthermore, in *ABCG2*-deficient mice, Aβ deposition increased significantly compared with that in wild-type mice after intravenous injection of Aβ, suggesting that ABCG2 can prevent Aβ from entering the brain [104]. In contrast with ABCB1 levels, ABCG2 gene and protein levels were upregulated in the cerebral vessels of AD patients and in the brains of mouse AD models [105]. The authors suggested that ABCG2 upregulation might act as a biomarker of cerebral amyloid angiopathy (CAA) vascular pathology. In addition, a genome-wide association study revealed the effect of ABCG2 C/C genotype absence or presence with apoE (APOE) on the risk of developing AD, which is a possible predisposing genetic factor for late-onset AD [106]. Some compounds that inhibit Aβ aggregation in AD models can penetrate the brain endothelium, mediated by ABCG2 [107].

### 4.3. ABCG4

ABCG4 is a half-transporter that always dimerizes with ABCG1 in order to become functional [108]. ABCG4 is expressed in brain capillary endothelial cells, glial cells and neurons and mediates the efflux of cholesterol to form apoE-containing lipoprotein [109, 110]. To the best of our knowledge, several studies have found that ABCG4 can affect Aβ production and Aβ clearance. Sano et al. reported that mature APP levels were increased in HEK/APPsw cells transiently transfected with ABCG4 compared with ABCG4-KM, which is a Walker A lysine mutant of ABCG4. Furthermore, the author demonstrated that the altered distribution of γ-secretase caused the reduced secretion of Aβ. They also demonstrated that when ABCG1 and ABCG4 were suppressed in SH-SY5Y cells, Aβ secretion was increased. These authors reasoned that ABCG4 could suppress Aβ production and Aβ plaque formation β[111]. Studies have reported the effect of ABCG4 on the clearance of Aβ from BBB. ABCG4 mediates the cellular efflux of Aβ in HEK293 cells stably transfected with mouse *abcg4*. Moreover, probucol inhibits Aβ efflux from HEK293-abcg4 cells completely [103]. Similarly, using an *Abcg4*-deficient mouse model, the author demonstrated that *abcg4* functions at the luminal surface of mouse brain capillary endothelial cells and can transport both Aβ and desmosterol. The author linked disordered sterol metabolism to competitive inhibition of Aβ efflux and progression of AD [110]. Furthermore, the study showed that ABCG4 levels are significantly upregulated in microglial cells and might contribute to Aβ degradation through phagocytosis [112]. Further studies are necessary to confirm the ABCG4 function in AD pathology and that ABCG4 may increase the clearance rate of Aβ for the prevention of AD.

### 4.4. ABCA1

ABCA1, also called cholesterol efflux regulatory protein (CERP), is widely expressed in brain tissues and can stimulate the efflux of cholesterol and phospholipid to ApoE [113]. Defects in cholesterol metabolism in the brain have been shown to be an important risk factor in AD pathogenesis because cholesterol levels are synergistic with Aβ production by affecting BACE1 [114]. ABCA1 is also expressed in brain capillary endothelial cells and neurons, but it does not transport Aβ directly [115]. ABCA1 can affect Aβ production and degradation rather than efflux transport across the BBB. The study showed that increased ABCA1 gene and protein levels induced by LXR ligands could increase secreted Aβ concentration and could be reversed by blocking ABCA1 expression using the RNAi method [116]. Furthermore, ABCA1 and ABCG1 regulates cholesterol homeostasis in BBB models*in vitro*. Bexarotene (an RXR agonist) induces ABCA1 expression, promotes cholesterol exchange between the blood and brain and decreases the influx of Aβ across the BBB [117]. Additionally, recent research found that high glucose downregulates ABCA1 expression and increases intracellular cholesterol levels, which regulates LXRα/ABCA1-mediated localization in the lipid raft and stimulates BACE1 in SK-N-MC cells [118]. These results suggest that ABCA1 is involved in the role of cholesterol and lipid rafts on APP processing by BACE1.

ABCA1 regulates levels of ApoE and ApoE lipidation, while ApoE is regarded as a chaperone for Aβ, affecting its clearance and aggregate [119]. In 12-month-old PDAPP Abca1^-/-^ mice, the Aβ level was found to be markedly higher than that in PDAPP Abca1^-/-^ mice without affecting APP processing, and carbonate-insoluble ApoE co-localized with Aβ plaques, implying that poorly lipidated ApoE co-deposits with insoluble Aβ [120]. The author further found that overexpression of Abca1 in the mouse brain increases ApoE lipidation and decreases Aβ deposition [121]. Similarly, ABCA1 reduction caused by microRNA-33 overexpression increases cellular cholesterol and Thioflavin S-positive plaques, leading to amyloidogenesis in mice [122]. However, in the hippocampal region of AD patients, ABCA1 mRNA expression is positively correlated with the severity of dementia [123]. Thus, affecting ABCA1 expression and its activity can be considered a therapeutic target to influence apoE/Aβ interactions in the brain. A recent study found that CS-6253, which can activate ABCA1 directly (ABCA1 agonist) *in vitro*, increased the lipidation of apoE4 and reversed apoE4-driven Aβ accumulation and tau hyperphosphorylation [113].

Some environmental and genetic factors could be the cause of late-onset AD. Dichlorodiphenyltrichloroethane (DDT) impairs the function of ABCA1 and further increases Aβ levels in H4-AβPPswe cells [124]. ABCA1 N1800H, a functional mutation found in 0.2% of individuals, was associated with a high risk of AD in the general population [125]. Furthermore, the ABCA1 rs2422493 (C-477T) polymorphism is statistically significantly associated with increasing AD risk in three genetic models [126]. In Han Chinese patients with AD, the ABCA1 rs2230806 polymorphism responded better to donepezil (DNP) treatment, which is a medication used to improve the cognition of patients with AD [127]. Similarly, the ABCA1 R219K K allele is a risk factor for lower ABCA1 in AD patients in northern China [128]. Based on the above results, the small molecular inducer P2X7 was found to enhance ABCA1 and ApoE without direct activation of the LXR pathway [129].

### 4.5. ABCA7

ABCA7, another CERP, shares 54% sequence identity with ABCA1 and is expressed in microglia, neurons, and brain endothelial cells [130, 131]. ABCA7 mediates the transfer of phospholipids and cholesterol across cell membranes to lipid-poor apolipoprotein acceptors [132]. Several groups have demonstrated that ABCA7 may regulate Aβ homeostasis and Aβ pathology. Studies have shown that ABCA7 plays a role in APP processing, leading to enhanced Aβ secretion, which may be linked to endocytosis activity in microglia [133]. In Chinese hamster ovary (CHO) cells stably expressing APP, ABCA7 could also significantly inhibit Aβ secretion without affecting the activities of α- and β-secretases [132]. In APP/PS1 mice, ABCA7 deficiency increases Aβ levels and exacerbates the amyloid plaque burden [134]. Consistent with these results, in transgenic mice, ABCA7 deficiency may aggravate the amyloid plaque burden in the brain [135]. ABCA7 is also involved in Aβ clearance in microglia. In Abca7^-/-^ mice, phagocytic Aβ clearance in microglia was found to be reduced significantly compared with that in wild-type mice [136]. Additionally, absent ABCA7 in mouse endothelial cells causes Aβ peptide reduction from basolateral-to-apical transport [137]. The author showed that ABCA7 affects Aβ transport not directly but in the presence of ApoI-J.

**Table 1:** An overview of the SLC and ABC transporters in the brain, with the proposed relevance to AD pathology.

Transporter	Location	Function	References
EAAT1/2	Expressed in astrocytes	Regulate glutamate homeostasis and affect excitotoxic neuronal damage in AD	[40]
GLUT1	Expressed in endothelial cell, neurons, astrocytes and microglia	Regulate glucose homeostasis in the brain Accelerate BBB breakdown, following secondary neurodegeneration caused by Aβ	[52] [60]
ABCB1	Expressed in endothelial cells, pericytes, astrocytes, the choroid plexus and neurons	Transport of endoxenobiotics across the encephalon Involved in the clearance of Aβ from the brain into blood ABCB1 at the BBB declines during normal aging	[83] [85] [90]
ABCG2	Highly expressed in BBB endothelial cells	Mediates the cellular efflux of Aβ in HEK293 cells	[103]
ABCG4	Expressed in brain capillary endothelial cells, glial cells and neurons	Mediates the efflux of cholesterol to form apoE-containing lipoprotein Suppresses ABCG1 and ABCG4 function and increases Aβ secretion Mediates the cellular efflux of Aβ in HEK293 cells stably transfected with mouse abcg4	[109, 110] [111] [103]
ABCA1	Widely expressed in brain tissues	Stimulates the efflux of cholesterol and phospholipid to ApoE Increased ABCA1 levels induced by LXR ligands can increase the secreted Aβ concentration Decreases the influx of Aβ across the BBB Regulates the levels of ApoE and ApoE lipidation ABCA1 rs2422493 (C-477T) polymorphism are associated with increasing AD risk	[113] [118] [117] [119] [126]
ABCA7	Expressed in microglia, neurons, and brain endothelial cells	Mediates the transfer of phospholipids and cholesterol across cell membranes Inhibits Aβ secretion without affecting the activities of α- and β-secretases Plays roles in APP processing, leading to enhanced Aβ secretion Phagocytic Aβ clearance in microglia is reduced in Abca7^-/-^ mice ABCA7 SNPs are relate to the occurrence of AD	[132] [132] [133] [136] [145]
ABCC1	Localized in brain endothelial cells, astrocytes and pericytes	Lack of ABCC1 could increase Aβ_40_ and Aβ_42_ levels compared to those in ABCC1-positive controls	[153]

Data from numerous genome-wide association studies (GWAS) have shown that *ABCA*7, the only ABC transporter identified by GWAS, is a risk factor for late-onset AD [138, 139]. GWAS have identified some gene variants that are considered to be established AD risk factors, such as *ApoE*, apolipoprotein J (*ApoJ*, clusterin) and phosphatidylinositol binding clathrin assembly protein (*PICALM*) [140, 141]. Both ApoE and ApoJ can affect Aβ clearance. Bell *et al* demonstrated that Aβ clearance in the BBB increased when ApoE bound to Aβ through LRP1-mediated transport, while ApoJ bound to Aβ through LRP2-mediated transport [142, 143]. The sequence of the ABCA7 SNP rs3764650 has been implicated in the occurrence of AD and is associated with a modest reduction in *ABCA7* expression [144, 145]. Furthermore, the *ABCA7* SNP (rs3764650) is also associated with AD risk in the Chinese population, while age and ApoE4 status could increase its risk [146]. *ABCA7* rs3764647 and *ABCA7* rs115550680 have been associated with AD risk in African Americans [147, 148]. *ABCA7* rs4147929 is associated with LOAD in the Spanish population [149].

### 4.6. ABCC1

ABCC1, a potent efflux pump at the BBB, is localized on both sides of the brain in capillary endothelial cells, astrocytes and pericytes [81, 150-152]. Several studies have demonstrated the involvement of ABCC1 in AD. *In vivo* studies showed that in APP/PS1 transgenic mice lacking ABCC1, Aβ_40_ and Aβ_42_ levels were increased compared to those of ABCC1-positive controls [153]. The authors further found that thiethylperazine, an activator of ABCC1, could reduce the Aβ burden in APP/PS1 transgenic mice ^[153]^. Similarly focused on ABCC1, Hofrichter et al provided the first specific evidence that St. John’s wort could increase ABCC1 export activity in the BBB and reduce Aβ levels, thereby attenuating Aβ-induced histopathology and alleviating memory impairments ^[154]^. These results suggest that St. John’s wort extracts might be a therapeutic drug for AD treatment, which necessitates further research.

### 4.7 Influence of AD progression on transporters

A neglected issue in the literature has been the influence of AD progression on changes in brain transporters. The course of AD treatment is divided into four stages: predementia and early, moderate, and advanced stages, according to the progressive pattern of cognitive and functional impairment. Aging is the primary risk factor in AD progression and can change transporter expression [155]. For example, studies found an age-related decrease in P-gp expression in normal aging rats, which can exacerbate the intracerebral accumulation of Aβ. However, accumulated Aβ can further downregulate P-gp expression [90]. Similarly, P-gp expression and activity are reduced in individuals with mild cognitive impairment and in AD patients, compared to normal individuals [156].

However, few researchers have focused on Aβ transporter expression as related to different signs and symptoms of AD, which might fortuitously be a new symptom marker in the early diagnosis of AD.

## 5. Conclusions and future perspectives

ABC transporters are involved in AD pathology directly or indirectly (Table 1). However, the molecular mechanisms have not been completely clarified and require further research. For instance, it is known that ABCB1 and ABCG2 are involved in Aβ clearance across the BBB. Unfortunately, their contribution ratio in the total Aβ clearance in the brain is still unknown. A better understanding of the mechanisms by which genetic variability in ABC and SLC transporters regulates Aβ production and degradation is needed. Furthermore, understanding the roles of ABC and SLC transporters in tau pathology and other risk factors in AD may help further explain the disease-modulating effects of the transporters. Therefore, exhaustively clarifying ABC and SLC transporter functions and expression location in the human brain is important for the development and optimization of viable therapeutic strategies that target transporters to combat neurodegenerative diseases.
